# The UJI Aerial Librarian Robot: A Quadcopter for Visual Library Inventory and Book Localisation

**DOI:** 10.3390/s21041079

**Published:** 2021-02-04

**Authors:** Ester Martinez-Martin, Eric Ferrer, Ilia Vasilev, Angel P. del Pobil

**Affiliations:** 1 RoViT, Department of Computer Science and Artificial Intelligence, University of Alicante, 03690 Alicante, Spain; 2 Robotic Intelligence Lab (RobInLab), Department of Engineering and Computer Science, Universitat Jaume I, 12071 Castellón, Spain; al312992@uji.es (E.F.); al365834@uji.es (I.V.); pobil@uji.es (A.P.d.P.); 3 Department of Interaction Science, Sungkyunkwan University, Seoul 110-745, Korea

**Keywords:** aerial robotics, library, unmanned aerial system (UAS), multi rotor, automated inventory

## Abstract

Over time, the field of robotics has provided solutions to automate routine tasks in different scenarios. In particular, libraries are awakening great interest in automated tasks since they are semi-structured environments where machines coexist with humans and several repetitive operations could be automatically performed. In addition, multirotor aerial vehicles have become very popular in many applications over the past decade, however autonomous flight in confined spaces still presents a number of challenges and the use of small drones has not been reported as an automated inventory device within libraries. This paper presents the UJI aerial librarian robot that leverages computer vision techniques to autonomously self-localize and navigate in a library for automated inventory and book localization. A control strategy to navigate along the library bookcases is presented by using visual markers for self-localization during a visual inspection of bookshelves. An image-based book recognition technique is described that combines computer vision techniques to detect the tags on the book spines, followed by an optical character recognizer (OCR) to convert the book code on the tags into text. These data can be used for library inventory. Misplaced books can be automatically detected, and a particular book can be located within the library. Our quadrotor robot was tested in a real library with promising results. The problems encountered and limitation of the system are discussed, along with its relation to similar applications, such as automated inventory in warehouses.

## 1. Introduction

Robotics is mainly focused on the automation of repetitive and tedious tasks. Since its beginnings, the main application domain was industrial settings due to their controlled nature. Over time, robotics has moved to more challenging situations taking place in human-centred environments. In this context, a library is a suitable environment since most of the librarian tasks are repetitive and time-consuming and they could be automated, such as inventory, relocation of missing or misplaced books, localization of a requested book, book retrieval, or reinstatement of a returned book on its corresponding bookshelf. Indeed, the automation of these tasks will result in a benefit in terms of cost and time. As a consequence, considerable research and development activity over the few last years has resulted in several technologies that try to automate these tasks, including librarian robots [1,2,3,4,5,6,7,8,9,10,11,12,13,14,15,16,17,18].

For the inventory task, a popular approach is to combine a robot platform with radio frequency identification (RFID) technology to quickly scan shelves in a similar way to warehouses [1]. This allows the robot to automatically register a misplaced book. Some examples are AdvanRobot [2]; AuRoSS (Autonomous Robotic Shelf Scanning) [3]; LibBot [4]; or TORY [5], a commercial solution for RFID inventory robot. However, this method can be too costly since all the books must be labeled and it requires RFID readers, antennas, and software license. It is also limited to this particular task, as discussed below.

For its part, computer vision has played a major role in the book recognition task. Early approaches aimed towards reading book titles on book spines. In [6], a control module placed a camera in a random position so that images were processed and regions were classified as book or non-book. Then, the book regions were segmented by using line segments and M-estimator SAmple Consensus (MSAC) based detection of dominant vanishing points, a process that is quite lengthy and complicated. Finally, adaptive Otsu’s thresholding was employed to extract book titles for recognition. The results show this approach is not sufficient in all cases and sometimes it fails to properly detect every bookshelf in the bookcase. Something similar happens with [7], where only one bookshelf is assumed to be visible. With the aim to suppress this restrictive constraint that cannot always be guaranteed, Tabassum et al. [8] proposed an approach for segmenting book spines and recognizing book titles. For that, the first step was to use horizontal edges to divide each image in bookshelves. Then, vertical edges were used to separate books, and the title characters were extracted by using bounding boxes and connected component regions. Among its drawbacks, we can mention the limited thickness of book spines, the required specific distance between the camera and bookshelf, and the particular orientation of the camera when the images are taken. As an improvement, we can cite the multi-stage framework for book detection presented by Jubair et al. [9] in which angular line detection is adopted to overcome the {vertical} book orientation. Even so, the book detection module results in a low success rate.

Alternatively, book detection can be considered as an object detection task. In this context, deep learning techniques that have been employed in recent years. Prashanth et al. [10] used the well-known convolutional network AlexNet to detect books in a library, while Zhu et al. [11] applied a faster Region-based Convolutional Neural Network (R-CNN), nevertheless, book recognition was not addressed by either of them. Finally, Yang et al. [12] proposed a revised version of the connectionist temporal classification (CTC) for reading text on the book spines.

With respect to book delivery, an example is the UJI librarian robot [13,14], an autonomous system assisting users from some book information introduced in the digital catalogue. From this information, the robot autonomously navigates to the corresponding bookshelf and localizes the book based on its code, extracting it from the bookshelf and delivering it to the user at the library desk. More recently, the National Library of Australia has incorporated Isaac, a robot coexisting with human staff in the underground storage to quickly provide the requested materials to the user (https://www.abc.net.au/news/2018-08-17/robot-couriers-set-efficiency-in-motion-at-national-library/10118356). Alternatively, teleoperation can also be used, as in [15,16]. Another solution is to combine a robot platform with RFID to localize a requested book within a bookshelf, as it is the case of Unnikrishnan et al. [17]. Here, after book localization, the robot sends a notification to the shelf unit that is in charge of activating the shelf tray so that the requested book is placed into the robot basket and delivered to the desk.

On the other hand, aerial robotics has grown to be a popular field in the last decade to the extent that unmanned aerial vehicles (UAVs)—or drones—have become a standard platform in the robotics research community [18] thanks to their versatility, high mobility, and ability to cover areas at different altitudes and locations. Indeed, this kind of robots have enabled a large variety of applications, such as traffic monitoring [19], tracking [20,21], surveillance [22], public protection and disaster relief operations [23], homeland security [24], film making [25], farming [26], mining [27], personal assistance [28,29], smart cities [30], or package delivery [31].

The main reason for the success of aerial robots lies in their advantages with respect to other robotic platforms. Among them, we can mention their current sufficient payload and flight endurance, improvements of battery, high maneuverability, small size, low inertia, safety, and low-cost. Compared with traditional wheeled robots, their main advantage is their ability to move in three-dimensional space with little effort, flying at different altitudes and hovering in the target area to collect precise information. This ability to move in 3D space, however, brings with it great scientific and technical challenges, specially in the case of autonomous flight in indoor spaces [32] for which perceptual intelligence based on aerial vision is called for to self-localize, navigate, and perform the desired tasks in human environments. Few such indoor systems exist, for instance, Harik et al. [33] describe an approach combining an unmanned ground vehicle (UGV) and an UAV for the automation of warehouse inventory. The UGV carries the UAV, which flies vertically but does not use aerial vision since it only employs an on-board frontal scanner to scan barcodes. This combination of UGV with UAV is also used to overcome the limited capacity of an UAV battery power, this shortcoming considerably restricts the working time and distance as well as the possible application scenarios of drones [34,35]. Alternatively, a swarm of UAVs can be used to cover the target area much more efficiently at the cost of an increased overall complexity of the system [36].

An additional problem is the need to keep the drone constantly moving during task execution, which significantly complicates the realization of inventory tasks [37]. In this sense, advanced localization technologies such as the global navigation satellite system (GNSS) and real-time kinematic (RTK) are widely used, however, they can only function correctly in an area with transmission coverage, which is specially critical when indoor environments are considered. Similarly, GPS sensors are not appropriate in indoors either due to the lack of signal.

As an alternative, computer vision technologies have been used for navigation of UAVs and usually vision information is processed in a ground station with robust algorithms [38]. This approach entails also problems with communications that may be interfered, and data transmission can turn slow due to the size and quantity of data. The use of maps is a state-of-the-art approach. Blosch et al. [39] employed a monocular camera with a visual SLAM algorithm such that the pose of the camera is tracked while, simultaneously, an incremental map of the surrounding region is being built. Nevertheless, some drifting problems occur forcing the system to be constantly corrected. For their part, Han et al. [40] proposed to use topological maps although they get a high accuracy, the processing does not work in real-time, making it inappropriate for our goal. An extended Kalman filter (EKF)-based multi-sensor fusion framework was used in [41] and in this case, a simple map consisting of pose information of attached tags is the base of a warehouse inventory application. Another common solution for UAV navigation is the use of landmarks. This is the case of Ibarra et al. [42], who used artificial landmarkers to provide navigation instructions, while estimating relative distance between the UAV and landmark. For that, a Jetson Nano Developer Kit from Nvidia was embedded in the UAV for image processing support. Note that particular illumination conditions are required for a good performance. Hummel et al. [43] have recently presented a distributed architecture for human-drone teaming focusing on timing and interaction issues, they illustrate the architecture with an algorithm for bookshelf scanning based on rectangle detection however, the experimental validation is not done with a real bookcase but only with a poster on which book mock-ups are depicted as one-color rectangles, separated by white gaps and with this set-up the authors report results consisting of the number of scanned books on the poster with a success rate of 50%. In summary, although a wide research in navigation has taken place (see [37,44,45,46] for additional relevant references), autonomous UAV navigation is still a challenge.

Recapitulating, ground-based robots have been and already are being used in libraries, but automation based on aerial robots have not been used so far due to the above-mentioned technological challenges. Our proposal is that an UAV can be used for library inspection leading to several advantages over human manual inspection such as saving time and cost, easy access to all bookshelves, reading several books per image, facilitating enhanced contextual awareness, and alerting to book misplacements in real-time. Even though a few inventory mechanisms based on drones are in place in warehouses, as mentioned above, to the best of our knowledge the use of drones has not been reported as an automated inventory device within libraries. In this paper, we present the UJI aerial librarian robot, an unmanned quadrotor drone—or quadcopter—that leverages computer vision techniques to autonomously self-localize and navigate within a library for automated inventory and book localization, identifying misplaced books. Our quadcopter robot has been tested in a real library with promising results. This paper is structured as follows: Section 2 describes the implemented approach for robot localization in a library, while the navigation procedure is introduced in Section 3; Section 4 describes the computer vision approach designed for book recognition; some experimental results are presented in Section 5, while some conclusions and future work are introduced in Section 6.

## 2. Localization

**Figure 1:**
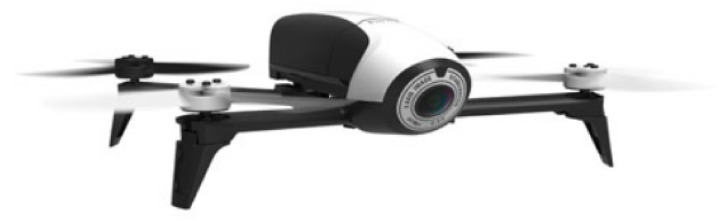
Parrot Bebop 2 quadcopter.

Even though a library typically presents a regular structure composed of systematically arranged bookcases, it is far from being perfectly organized and optimized due to both the dynamic conditions of library operation and the human factor. So, a robot system capable of going through all the library racks is required. Since some bookcases may be high and wide, ground robots (e.g., wheeled platforms, legged robots, or manipulators on a mobile platform) could have problems to reach all their bookshelves without external help. As a solution, an UAV can be used. Due to its recent popularity, there are several types of these vehicles in the market. Among them, a copter looks like the most appropriate for using in indoor facilities as it does not need too much space to operate and can easily hover at one point or move with low speed (a requirement to properly identify each book on the shelf). In particular, we used the Parrot Bebop 2 quadcopter [47] (see Figure 1). This commercial drone has a size of 28×32×3.6 cm, a battery of around 20 min of flying durability, and an RGB camera able to take pictures with a 4096 × 3072 resolution, although a maximum of 856 × 480 pixels is allowed when real time is required.

**Figure 2:**
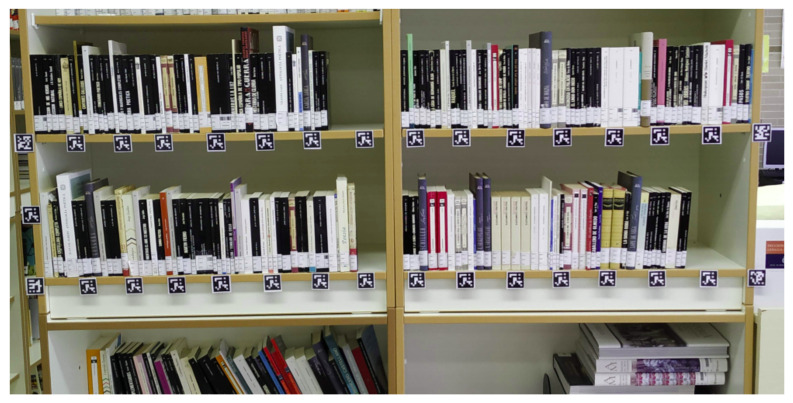
A bookcase sample equipped with ArUco markers.

Although a GNSS sensor is typically used for outdoor localization, GPS information is not precise enough when flying indoors. As a consequence, and based on the study presented in [48] and the localization quality and cost of the existing methods, we used a self-tracking approach based on visual markers in order to compute the quadcopter position in the library. To this end, several ArUco markers [49] were placed on the bookshelves in specific poses (see Figure 2) so that the quadcopter pose is estimated according to the number and type of visible markers.

**Figure 3:**
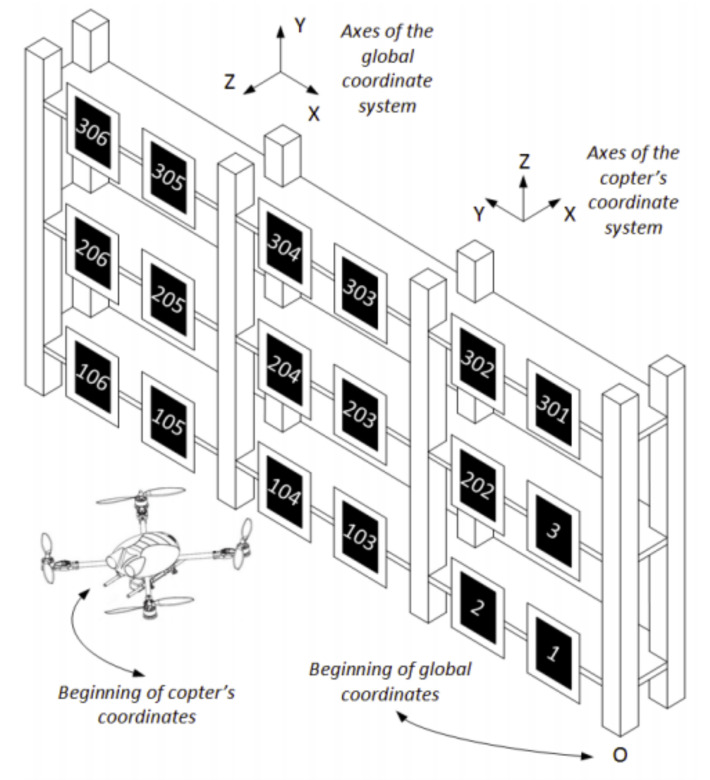
Pattern for placing the markers on the bookcase based on their index numbers. Note also the orientation of the two coordinate systems.

Note that the precision of the quadcopter pose estimation directly depends on the knowledge about the markers pose, since the camera is rigidly mounted on the drone and its pose with respect to it is usually constant and known. However, although the markers are placed at specific positions, their placement can be done manually and without any special devices to ensure the precision of the positions of the markers. Consequently, significant errors of up to several centimetres can be introduced into the real positions of the markers with respect to their planned positions. Therefore, it is necessary to introduce a calibration process. With that aim, visual information from the camera is used to calculate mutual poses of the markers as follows:

1. The three markers closest to the origin of the global coordinate system (let us call them *initial markers*) are chosen, manually measured, and preset their poses in global coordinates in a Cartesian coordinate system that we arbitrarily set up (see Figure 3);
2. Mutual poses of the initial markers are searched and, from that information, their preset global poses are corrected so as to not contradict camera-measured mutual poses;
3. On the basis of corrected poses of the three initial markers, positions of all other present markers are obtained.

Mutual marker poses can be obtained because the marker-tracking library (OpenCV [50] in our case) provides the pose in the camera coordinate system for each marker in every frame. So, if two markers, with index numbers A and B, are present in a frame, two three-component position vectors $P_{C}^{M_{A}}$ and $P_{C}^{M_{B}}$, and two orientation quaternions $Q_{C}^{M_{A}}$ and $P_{C}^{M_{B}}$ (in camera coordinates) are obtained. From these four vectors, transformation matrices $T_{C}^{M_{A}}$ and $T_{C}^{M_{B}}$ are derived according to Equation (1).

(1) $$T_{C}^{M}=\left[\begin{array}{cc} R_{C}^{M} & P_{C}^{M}\\ 0 & 1 \end{array}\right]$$

where $R_{C}^{M}$ is the rotation matrix obtained from the orientation quaternion $Q_{C}^{M}$ following Equation (2) as defined in [51].

(2) $$R_{C}^{M}=\left[\begin{array}{ccc} 1-2y_{C}^{M^{2}}-2z_{C}^{M^{2}} & 2x_{C}^{M^{2}}y_{C}^{M^{2}}+2w_{C}^{M^{2}}z_{C}^{M^{2}} & 2x_{C}^{M^{2}}z_{C}^{M^{2}}-2w_{C}^{M^{2}}y_{C}^{M^{2}}\\ 2x_{C}^{M^{2}}y_{C}^{M^{2}}-2w_{C}^{M^{2}}z_{C}^{M^{2}} & 1-2x_{C}^{M^{2}}-2z_{C}^{M^{2}} & 2y_{C}^{M^{2}}z_{C}^{M^{2}}+2w_{C}^{M^{2}}x_{C}^{M^{2}}\\ 2x_{C}^{M^{2}}z_{C}^{M^{2}}+2w_{C}^{M^{2}}y_{C}^{M^{2}} & 2y_{C}^{M^{2}}z_{C}^{M^{2}}-2w_{C}^{M^{2}}x_{C}^{M^{2}} & 1-2x_{C}^{M^{2}}-2y_{C}^{M^{2}} \end{array}\right].$$

The next step is to find the camera pose in the marker coordinate system through the inverse matrix [52]:

(3) $$T_{C}^{M^{-1}}=\left[\begin{array}{cc} R_{C}^{M^{T}} & -R_{C}^{M^{T}}\cdot P_{C}^{M}\\ 0 & 1 \end{array}\right].$$

Thus, the transformation matrix for conversion from the reference reference of one marker into the reference frame of the other marker is obtained as follows:

(4) $$T_{M_{A}}^{M_{B}}=T_{M_{A}}^{C}\cdot T_{C}^{M_{B}}.$$

Then, $T_{M_{A}}^{M_{B}}$ is divided into $P_{M_{A}}^{M_{B}}$ and $Q_{M_{A}}^{M_{B}}$. With the purpose of getting a higher precision in mutual pose estimation, several images of the same pair of markers are taken from different points of view and the average is considered for quadcopter localization.

This process is performed for each appropriate pair of markers when more than two markers are present in the frame. The markers are sorted in ascending order according to their index numbers, which are all different. So, as illustrated in Figure 3, the index number of markers increases as markers get placed further from the origin of the global coordinate system. In addition, the index numbers of the markers within the same shelf increase by one for every consecutive marker moving away from the origin of the global coordinate system along the same shelf (that is, from the beginning to the end of the shelf). Similarly, the index numbers increase by one hundred for every consecutive marker in the same column from the lowest shelf to the uppermost shelf. Note that this convention is not followed by three markers close to the origin of the global coordinate system. The reason lies in the improvement of the quadcopter pose estimation so that, when the poses of those three initial markers are known with enough precision, locations of all other markers placed on the shelving can also be automatically detected with the help of the camera. Bundle adjustment is used for the three initial markers and poses for every of the “further” markers are calculated as an average based on the positions obtained from several “nearer” markers. An additional method to increase the stability of localization with respect to the robot map is used—it is a weighted average of the pose considering several markers falling into the field of view.

## 3. Navigation

As soon as the information about the quadcopter pose with respect to the bookshelf is known, it is necessary to define and implement the proper scrutiny path by setting appropriate velocity of the robot movement at every moment of time. Note that the possibility to control the velocity of the quadcopter (i.e., linear X speed, linear Y speed, linear Z speed, and angular yaw speed) is implicitly presented by software-hardware implementation of Parrot Bebop 2. In addition, the quadcopter has difficulties indoors to keep the same position for a long time when no movement commands are given.

**Figure 4:**
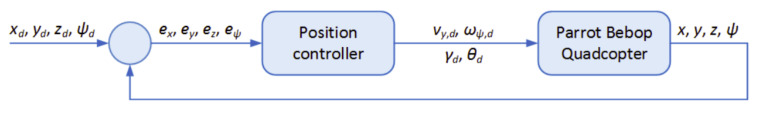
Flowchart of our implemented position control system where *x*, *y*, *z*, and ψ represent the actual pose of the quadcopter; $x_{d}$, $y_{d}$, $z_{d}$, and $\psi_{d}$ represent its current desired pose; $e_{x}$, $e_{y}$, $e_{z}$, and $e_{\psi }$ represent the error between the actual and the desired pose of the quadcopter; and $v_{y,d}$, $\gamma_{d}$, $\theta_{d}$ and $\omega_{\psi ,d}$ represent the commands sent from the controller to the quadcopter.

**Figure 5:**
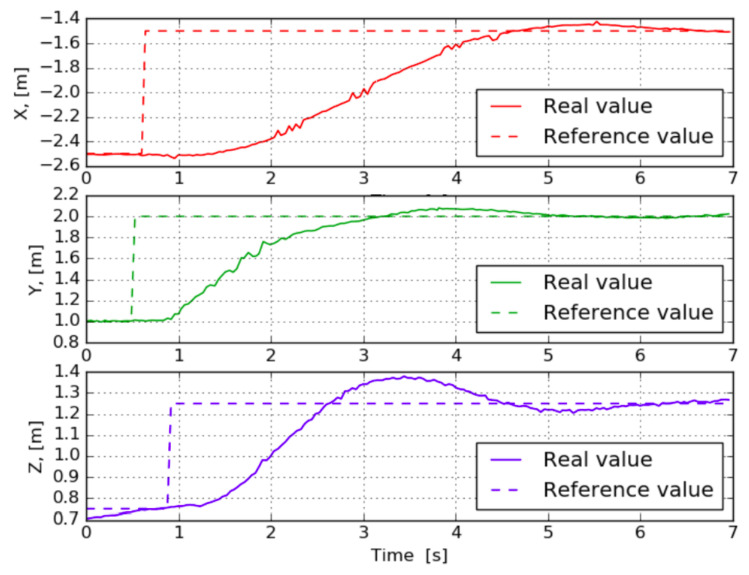
Transition processed for controlled change of quadcopter position during flight when the Parrot Drone (PD) controller is used. Graphs for the three axes are independent and represent different moments in flight. For each graph, when transition processing is taking place, reference values for the other axes remain constant.

Consequently, it is required to continuously adjust the movement commands based on the position feedback. First, a position control module is in charge of ensuring that the quadcopter is moving towards the goal point adequately, or that it does not drift away from the goal point when it is already there. For that, it estimates and sends the Y linear translation ($v_{y,d}$), roll rotation ($\gamma_{d}$), pitch ($\theta_{d}$), and yaw ($\omega_{\psi ,d}$) angles to the quadcopter (see Figure 4). So, proportional control can be applied to control Y coordinate and yaw angle of the quadcopter in accordance with Equation (5).

(5) $$\begin{array}{c} v_{y,d}\left(t\right)=K_{P,y}\cdot e_{y}\left(t\right)\\ \omega_{\psi ,d}=K_{P,\psi }\cdot e_{\psi }\left(t\right) \end{array}$$

where $K_{P,y}$ and $K_{P,\psi }$ are predefined proportional coefficients, while $e_{y}\left(t\right)$ and $e_{\psi }\left(t\right)$ represent the errors between the actual and desired quadcopter in terms of the corresponding coordinate. When a similar principle was considered with roll and pitch angles to control X and Z coordinates, unsatisfactory results were obtained. The main reason is that X and Z quadcopter speeds are not proportional to roll and pitch angles, as it can be derived from a simple dynamical model of the quadcopter [53]. Instead, there is a dynamical connection between the values and it is possible to take this into account by adding a differential component to the controller. Hence, the desired values for pitch and roll angles can be calculated according to Equation (6), obtaining more accurate results as shown in Figure 5. Note that, as seen in Figure 3, the Z axis is the global horizontal axis that is expected to be aligned with the longitudinal axis (X) of the quadcopter, so the position of the quadcopter on this axis is exactly expected to be manipulated by changing its pitch angle, i.e., with a rotation around the copter’s Y axis and similarly for the other pairs of global and quadcopter axes. The reader is referred to [53,54] for a more detailed rationale behind Equation (6).

(6) $$\begin{array}{c} \gamma_{d}\left(t\right)=K_{P,x}\cdot e_{x}\left(t\right)+K_{D}\cdot \frac{de_{x}\left(t\right)}{dt}\\ \theta_{d}\left(t\right)=K_{P,z}\cdot e_{z}\left(t\right)+K_{D}\cdot \frac{de_{z}\left(t\right)}{dt} \end{array}$$

**Figure 6:**
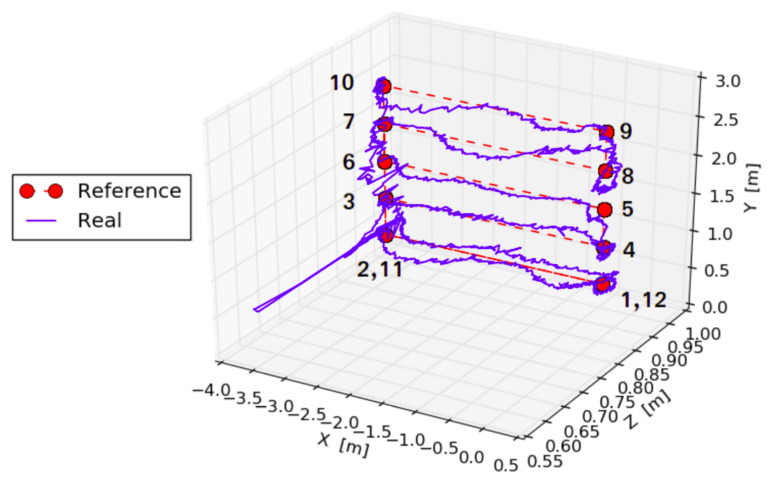
Experimental trajectory of the Parrot Bebop 2 with 12 waypoints located along the bookcase in the global coordinate system. Significant deviation from the straight path on the Z coordinate in the lower part of the trajectory is due to errors in video link and loss of orientation of the quadcopter.

Once the quadcopter is able to reach the desired points in 3D space, the next step is path planning. In this case, the quadcopter should fly along each shelf following a linear path. As illustrated in Figure 6, the followed trajectory departs from the straight line, although all the waypoints are reached successfully. Thus, several experiments were carried out to determine the appropriate number of waypoints so that the bookshelves are completely covered. In our case, 8 waypoints for a shelf are required.

### Visiting a Sequence of Bookcases

So far, we focused on one bookcase since this is where the real novelty is for automated book inventory and localization using aerial vision. Moving within the aisles of a regular structure has already been addressed in the literature [41], this is a common behavior whenever a drone has to fly along the aisles limited by sets of racks or walls. To this end, apart for moving to a specific point at a bookcase, a behavior to move to a point outside a bookcase, along with a transition between the bookcases, and a plan to visit the bookcases one after the other in a predefined sequence, need to be in place. A detailed description of these procedures goes beyond the purpose of this paper. They are based on an extension of the techniques for navigation and localization that are described above.

Essentially, it is an iterative process made up of three steps: Move the drone to the starting bookcase, scan the bookcase, and move the drone to the next bookcase following a predefined trajectory with a standard path planning algorithm with via points. Additional visual tags are placed in the area between the bookcases or on the sides of the bookcases, which also include the identification of the bookcase. As a previous step, a model of the library is built, in which the physical layout is mapped to the logical structure, including the position of each bookcase inside the library map.

The above procedures can be implemented as a classical three-layer architecture with the lowest layer (reactive controller) implementing different real-time feedback behaviors, middle layer (sequencer) switching the behaviors one by one and supplying parameters to them based on the current situation, and the top layer (deliberative planner) arranging the overall plan for the sequencer based on the specific goals.

## 4. Book Recognition

With the aim to perform librarian tasks, the quadcopter must be able to recognize each book within its field of view. For that reason, an analysis of the visual features to properly identify each book is required. However, there are a wide range of visual features that vary from one book to another such as size, thickness, color, and title style. This makes book recognition a difficult task to achieve.

In this context, deep learning (DL) techniques could be considered since they provide classifiers that can learn visual features. These techniques perform automatic feature extraction from raw data, directly classifying all the visual elements in the taken image. For that, a large set of labeled data is necessary, that is, hundreds of images taken from different points of view, orientations, and scales are required for each book to be learned. Due to the great amount of books in a library, this process and its corresponding training stage would be highly time-consuming and unfeasible.

As mentioned above, RFID systems could be an alternative. However, its main drawback is that all the books in the range of the RFID reader will be recognized, without providing information about the exact location of each book, and limiting the librarian robot tasks to just inventory. In addition, attaching an RFID tag to all the books would result in a very time consuming processand this would also be the case for other alternatives such as affixing QR codes to the book spines, and it is for this reason that they were also discarded.

Instead, our approach takes advantage of the ordinary tags for book cataloging that are used in most of the libraries worldwide. Basically, a tag is a homogeneously colored label where an alphanumeric code according to the library catalog is written in black. These tags are usually placed at the bottom of the book spines. On this basis, we have designed a novel aerial vision approach for book recognition that is composed of the detection of the book tags, followed by the book code (or signature) on each tag by means of the optical character recognition (OCR).

### 4.1. Book Tag Detection

**Figure 7:**
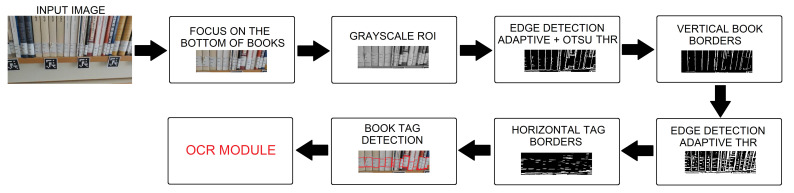
Flowchart of our vision approach for book tag detection.

With the aim of detecting a tag for each book in the image, the first step is to automatically define a region of interest, that is, focus the search in the area where the books are instead of the whole image. For that, the ArUco markers are used. All the ArUco markers included in the considered dictionary are searched in each frame so that we obtain a list of all the 2D corner positions corresponding to each detected marker together with its identifier. From these data, a horizontal line joining the top marker corners is defined. This line represents the base of the considered region of interest, which is 200 pixels high, as illustrated in Figure 7. The resulting image is converted to grayscale as follows:

(7) gray=0.299∗R+0.58∗G+0.114∗B.

Next, the image is filtered in order to remove noise. In particular, a 9 × 9 Gaussian kernel is used. The subsequent stage corresponds to edge detection and for that, a threshold for each region of the image is estimated by means of a combination of binary thresholding with the Otsu thresholding. The use of local thresholds instead of a global one for the whole image provides better segmentation results for images with varying illumination, which is a common situation in a library. Then, an erosion operation allows the system to extract the vertical lines identifying the book borders. Similarly, an adaptive thresholding followed by a pair of morphological operations allows one to identify the horizontal edges of the book tags.

Once all the edges have been properly distinguished, the intersection points are searched. Finally, the book tags are obtained from those intersection points. Indeed, superimposing the lines on the original image confirmed the detection of book tag boundaries.

**Figure 8:**
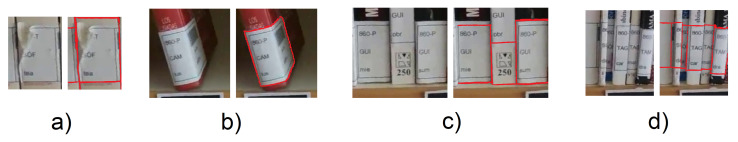
Some results of the book tag detection illustrating successful results for partially broken (**a**) and slanted tags (**b**), and problematic cases such as too high tags (**c**) and unclear vertical separation (**d**).

Some of the obtained experimental results are shown in Figure 8. As illustrated, the designed approach properly detects the book tags in most of the cases, even when they are partially broken or the book is inclined. However, the approach fails when the book tag is not placed at the bottom of the book spine, as in the case of Figure 8c. Another error takes place when two consecutive books are too similar and there is no clear vertical space between them, since vertical lines are not accurately extracted (see Figure 8d). As an overall result of several runs, the obtained average book recognition rate is 72% when all individual frames are considered. Since often a tag that is incorrectly identified in one frame is successfully detected in the next overlapping frame, the actual success rate is that 85% of the book tags in the bookshelves are correctly detected.

### 4.2. Optical Character Recognition (OCR)

OCR converts a 2D text image into machine-encoded text (i.e., string chains). This approach has been widely used in the last few decades to provide automated text entry into a computer instead of typing all the text [55,56]. Recent OCR engines make use of deep learning techniques to improve their accuracy in text detection and recognition. An example is *Tesseract* [57], a very popular open-source OCR engine and one of the first approaches to obtain good recognition accuracy results [58]. Basically, the input image is processed in boxes (rectangles), line by line, feeding into a long short term memory (LSTM) model.

**Figure 9:**
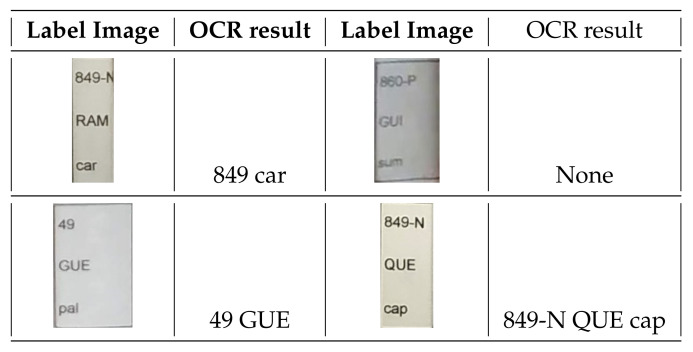
Some results of optical character recognizer (OCR) label recognition.

In this work, Pytesseract, a wrapper for Tesseract-OCR Engine, was used. Each tag detected by the book tag detection module is fed into Pytesseract for its processing (see Figure 9 for some results). Then, the generated output is checked in terms of its consistency, since only partial text could have been provided due to broken book tags or the book thinness. In addition, skewed or rotated text may also make the OCR fail. With all this, the obtained average of book code recognition is 75%, which means that 75% of the books in the bookshelves were detected correctly. Note that the recognition success may drastically drop to 25% when the image resolution is poor. The reason lies in the loss of visual information on the labels and, as a consequence, the OCR is not able to properly recognize the text on the detected labels.

## 5. Experimental Results

**Figure 10:**
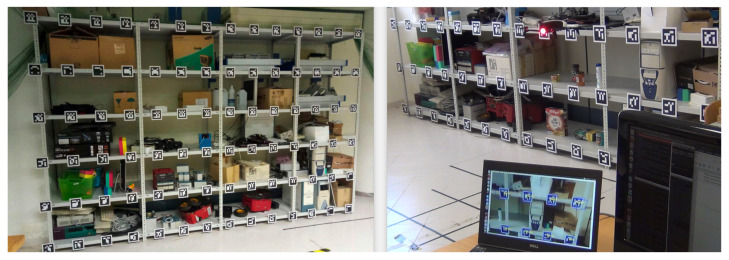
Preliminary experiments were conducted at UJI RobInLab to test localization and navigation.

With the aim to evaluate the performance of the developed system, first preliminary experiments were conducted at UJI Robotic Intelligence Laboratory in order to test the localization and navigation approach in which instead of books, the shelves contained a variety of ordinary objects (Figure 10). The results were satisfactory and the system was able to deal with issues such as drift and pose recovery. Then, the UJI aerial librarian robot was tested in the library of the high school I.E.S. Els ports. Experiments were conducted outside opening hours due to the noise and propeller wash associated with the quadcopter in flight in the otherwise silent atmosphere of a library.

In the experiments, the robot navigated around wooden bookcases with a height of 2.4 m, a width of 1.7 m, and a depth of 40 cm (see Figure 2). Each bookcase is composed of six bookshelves. Each bookshelf was equipped with several visual 7 × 7 ArUco markers necessary for localization and navigation. In particular, they were attached to the front side of each shelf, with a horizontal distance between the markers of some 7 cm. Such placement resulted in 8 markers per shelf and a total of 48 markers per bookcase.

**Figure 11:**
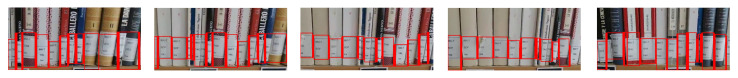
Some results of book recognition through a bookcase.

After the experimental set-up, the gathering of book information (i.e., global localization and book recognition) was run for several times. Several conditions were considered such as misplaced books, occlusions, different positions in depth, and so on. These experiments highlight the importance of precise and quick robot localization to properly identify all the books in a bookcase. In addition, the capture of several images from the same position usually improves the results of book recognition. As overall result, the average rate of book recognition and localization was 65%, which means that 65% of the books in the bookcase were detected correctly (see Figure 11). Despite its general feasibility, the false detection rate of 35% still implies a considerably inventory loss, that would require a refinement of the approach for future real-world application.

## 6. Conclusions

Keeping an organized and well classified library is a complex task given its dynamic nature. In consequence, inventory, book localization, and replacement of misplaced books are required on a regular basis. In this context, robotics can be instrumental. In particular, the 3D maneuverability of a small aerial robot can be of great interest since it could easily reach all the bookshelves, regardless of their position in space. In this paper, we presented the first robot of this nature, the UJI aerial librarian robot, an autonomous quadcopter that is able to globally localize books in an ordinary library by leveraging an aerial vision approach for localization, navigation, and book recognition.

To this end, the first step was to design a procedure to globally localize the robot within a library. For that, visual ArUco markers were suitably attached to the bookshelves so that the robot was able to estimate its position from the mutual marker poses. Then, a study about the robot navigation resulted in a path planning approach based on waypoints for each bookshelf. The next step corresponds to book recognition. For that, we took advantage of the tags on the book spines themselves, which are used for cataloging. Thus, the taken image is cropped, focusing on the bottom part of the books. After that, a new aerial vision approach was used to properly detect all the book tags in the image. Later, each book tag was cut and fed into the OCR module to extract and identify the characters composing the book code on the tag. Finally, the obtained code was checked in terms of catalog format, discarding the invalid ones. This failure situation can happen when the book code is damaged, the book spine is too thin, the book tag was unsuccessfully isolated, or the characters are too skewed.

Our experimental study was conducted in a real scenario (a high school library) with two bookcases composed of six bookshelves each, as partially shown in Figure 2. Our results revealed the need to take several images from the same position as well as to obtain precise and quick robot localization in order to properly identify all the books in a bookcase. After several tests with varying conditions, the accuracy rate was 65%. This result was obtained from a visual analysis of two bookshelves with a total of 88 books on it. If we do not take into account the books with unreadable tags (due to damaged tags or too thin book spines) – since not even humans can read them – this rate increases to 72%. We believe these are very promising results, for a first ever proof-of-concept system, that opens the way to further research along this path.

In addition, this work improves on state-of-the-art approaches to other applications of UAVs. First, our ad-hoc control strategy for autonomous flight is aimed at overcoming two main problems: An automated collision-free navigation in an indoor environment avoiding interference such as Wi-Fi, magnetic fields, telephony, or radio signals and an accurate book inspection through the different bookcases of a library, while a continuous motion takes place. Then, although there is a wide literature on book detection and recognition, this is still an open issue due to its inherent difficulty. In this sense, our approach combines different computer vision algorithms to accurately detect and extract book tags for recognition. In this case, our contributions pertain to the detection and recognition of books with different visual features (e.g., color and size), as well as the suppression of requirements such as specific distance between camera and book, book orientation, or camera orientation with respect to the bookshelf.

Still, we acknowledge that there is plenty of room for further improvements in the methods employed in our prototype. In the case of localization, using multiple well separated points (instead of averaging of poses estimated using single markers) may improve the performance of the system, and more exploration could be done on the possibility of model-based filtering in order to obtain better estimations of robot poses (which turned out to be difficult for the Parrot Bebop due to the particularities of its internal control loop). Since the current inventory loss would also be excessive for permanent deployment in a library, further research is needed to improve the book recognition module. Part of the problem is the available speed and resolution of the built-in camera mounted on the low-cost drone used, of which higher quality visual sensors are obviously required for better performance. Additional improvements in the stabilization of the quadcopter would help avoid somewhat blurred characters in the tag images, along with more elaborated algorithms to process the sequence of images of the same book captured while moving, so that ad-hoc context-aware OCR modules can better identify characters composing the book codes on the tags.

Even so, since books with erroneous tag readings can be localized in images, this information can be incorporated in the inventory report so that only those books would need to be checked manually. In any case, this should be done whenever unreadable tags are present (damaged, too thin books, etc.) and the book has to be taken out of the shelf for the tag to be properly read, or to look at the code inside the book or on the back cover. Thus, for a 65% success rate, we estimate that the inventory time would be reduced to a 42% of the fully manual time cost, considering that the required time is around one order of magnitude smaller for the UJI Aerial Librarian Robot.
